# 

**Published:** 1831-11

**Authors:** 


*** *Communications have been received from* Mr. Edwin Long, Mr. Laidlaw, Mr. Taylor, Mr. Guthrie, &c.

